# Polystyrene Microplastics Induce Sustained Cardiovascular Redox Imbalance and Alter Mitochondrial Quality Control

**DOI:** 10.3390/antiox15070816

**Published:** 2026-06-29

**Authors:** Ting-Yu Tsai, Pei-Hsuan Lu, Eddy Owaga, Yi-Sheng Tsai, Chia-Wen Chen, Rong-Hong Hsieh

**Affiliations:** 1 School of Nutrition and Health Sciences, College of Nutrition, Taipei Medical University, Taipei 11031, Taiwan; 2 Institute of Food Bioresources Technology, Dedan Kimathi University of Technology, Nyeri 10143, Kenya; 3 Research Center of Nutritional Medicine, College of Nutrition, Taipei Medical University, Taipei 11031, Taiwan

**Keywords:** redox imbalance, NOX4, mitochondrial dysfunction, mitochondrial respiratory chain, mitochondrial biogenesis, mitophagy

## Abstract

Microplastic exposure is an emerging environmental risk factor for cardiovascular health; however, whether cardiovascular alterations can be detected after exposure cessation remains unclear. This study investigated subclinical cardiovascular alterations following repeated oral exposure to polystyrene microplastics (PSMPs), with particular emphasis on redox imbalance and mitochondrial function in delayed cardiovascular alterations. Male Sprague-Dawley rats were administered 0.5 μm PSMPs via oral gavage at varying dosages of 5 or 20 mg/kg every 5 days for 70 days, followed by a 35-day exposure-free period. Repeated exposure to PSMPs did not affect body or organ weights but altered cardiac serum biochemical markers. Cardiac tissue exhibited elevated NADPH oxidase 4 (NOX4) expression and decreased superoxide dismutase 1 (SOD1), SOD2, and catalase (CAT) activities, whereas malondialdehyde (MDA) levels remained unchanged, indicating a state of chronic, low-level oxidative stress. Mitochondrial respiratory chain activities, including nicotinamide adenine dinucleotide cytochrome c reductase (NCCR) and succinate cytochrome c reductase (SCCR), were significantly reduced. Ultrastructural analysis revealed mitochondrial swelling and cristae disruption. In parallel, mitochondrial biogenesis-related proteins, including peroxisome proliferator-activated receptor gamma coactivator-1 alpha (PGC-1α), nuclear respiratory factor 1 (NRF-1), and mitochondrial transcription factor A (TFAM), were downregulated, while mitophagy markers, including PTEN-induced kinase 1 (PINK1), Parkin RBR E3 ubiquitin protein ligase (Parkin), microtubule-associated protein 1 light chain 3 (LC3), and sequestosome 1 (p62), were upregulated. Notably, most significant alterations were primarily observed in the high-dose group. Furthermore, the aorta showed increased oxidative stress markers without overt structural remodeling. These findings suggest that repeated exposure to PSMP is associated with subclinical cardiac redox–mitochondrial dysregulation, potentially involving redox imbalance, impaired mitochondrial respiratory chain activity, reduced mitochondrial biogenesis, and altered mitochondrial quality-control markers.

## 1. Introduction

Polystyrene microplastics (PSMPs), generated from the fragmentation and environmental weathering of plastic products, have become ubiquitous environmental pollutants and are increasingly detected in human food and drinking water. Microplastics and nanoplastics have been identified in bottled water, seafood, salt, and other processed foods, raising concerns about long-term, low-dose oral exposure in the general population. It has been estimated that humans may inadvertently ingest approximately 0.1–5 g of microplastics per week through multiple exposure pathways, although these estimates vary widely depending on the mode [1]. In addition to dietary sources, plastic particles have also been detected in human blood, placenta, feces, and other biological samples, indicating their ability to enter systemic circulation and reach internal organs [2,3]. These observations suggest that ingested microplastics are capable of reaching internal organs and potentially exerting systemic biological effects.

A growing body of evidence from human studies further suggests that microplastics and nanoplastics can access the cardiovascular system. Plastics derived from multiple polymer types have been detected in cardiovascular tissues, including the pericardium, epicardial adipose tissue, myocardium, and left atrial appendage, suggesting that circulating plastic particles can reach the cardiac microenvironment [3,4]. The heart is particularly susceptible to oxidative stress due to its exceptionally high mitochondrial content and reliance on oxidative phosphorylation. Microplastic exposure can increase reactive oxygen species (ROS) production and inhibit endogenous antioxidant defenses, leading to redox imbalance in mitochondria-rich organs such as the heart [5,6]. Excessive ROS can impair electron transport chain function, disrupt mitochondrial cristae, and compromise adenosine triphosphate (ATP) production [7,8,9].

Among ROS-generating systems, NADPH oxidase 4 (NOX4) has emerged as a key redox regulator with close links to mitochondrial function. NOX4 is constitutively active and predominantly generates hydrogen peroxide, and has been reported to localize to mitochondria where it can interact with respiratory chain components and influence mitochondrial bioenergetics. NOX4 can influence mitochondrial bioenergetics and redox homeostasis through pathways involving peroxisome proliferator-activated receptor gamma coactivator-1 alpha (PGC-1α) and nuclear factor erythroid 2-related factor 2 (Nrf2) [10].

Mitochondrial integrity is further maintained by an integrated mitochondrial quality-control (MQC) network encompassing mitochondrial biogenesis, selective removal of damaged mitochondria via mitophagy, and mitochondrial dynamics (fusion and fission). Mitochondrial biogenesis is transcriptionally regulated by PGC-1α and downstream nuclear factors, including nuclear respiratory factor 1 (NRF-1) and mitochondrial transcription factor A (TFAM), which coordinate mtDNA replication and organelle renewal. Conversely, PTEN-induced kinase 1 (PINK1)/Parkin RBR E3 ubiquitin protein ligase (Parkin)-dependent mitophagy selectively targets dysfunctional mitochondria for autophagic degradation through microtubule-associated protein 1 light chain 3 (LC3)-mediated pathways [11,12]. Disruption of MQC has been closely associated with pathological cardiac remodeling and heart failure, highlighting mitochondrial quality control as a potential mechanistic link between microplastic-induced oxidative stress and cardiac injury [13,14].

Importantly, while the heart is highly vulnerable due to its dense mitochondrial content, large elastic arteries such as the aorta are also continuously exposed to circulating microplastics and oxidative stress. However, due to distinct cellular compositions, metabolic demands, and structural properties, cardiac and vascular tissues may exhibit divergent mitochondrial and redox responses to plastic particle exposure.

Despite mounting evidence linking PSMPs exposure to oxidative stress and mitochondrial dysfunction, most studies have focused on continuous exposure models, leaving a limited understanding of whether cardiovascular mitochondrial alterations persist after exposure cessation. Whether PSMPs-induced mitochondrial perturbations represent transient adaptive responses or long-lasting pathological reprogramming remains unresolved. Therefore, the present study aimed to determine whether repeated oral exposure to PSMPs induces long-term alterations in cardiac mitochondrial function following exposure cessation. In this study, rats were subjected to a 70-day PSMPs exposure protocol followed by a 35-day observation period, and cardiac redox status, mitochondrial respiratory chain activity, ultrastructural integrity, and mitochondrial quality control were evaluated. Overall findings demonstrate that PSMPs’ exposure is associated with delayed cardiac redox imbalance and mitochondrial dysfunction.

## 2. Materials and Methods

### 2.1. Microplastics and Characterization

PSMPs with a particle diameter of 0.5 μm were obtained from Bangs Laboratories, Inc. (Fishers, IN, USA, Cat. No: PS03001). Prior to gavage, the particles were suspended in sterile distilled water at the desired concentrations. The suspension was sonicated for 10 min immediately before administration to ensure uniform dispersion. Particle morphology was examined using transmission electron microscopy (TEM; HT-7700, Hitachi, Tokyo, Japan). Hydrodynamic particle size distribution and zeta potential were analyzed using a Zetasizer Nano ZSP (Malvern Panalytical, Malvern, UK) in sterile distilled water.

### 2.2. Animals and Experimental Design

All experimental procedures were approved by the Institutional Animal Care and Use Committee of Taipei Medical University (IACUC No. LAC-2024-0424) and conducted in accordance with institutional animal care guidelines.

Male Sprague-Dawley rats (5 weeks old; weighing 140–150 g; *n* = 6 per group) were obtained from BioLASCO (Taipei, Taiwan) and housed under controlled environmental conditions (22 ± 2 °C, 12 h light/dark cycle) with ad libitum access to food and water.

After a 7-day acclimation period, animals were randomly assigned to three groups: control (C, received ultrapure water), low-dose PSMPs (L, received 5 mg/kg PSMPs, selected based on previously reported PSMP exposure levels and environmentally relevant exposure estimates [1], and high-dose PSMPs (H, received 20 mg/kg PSMPs, selected within the range of previously reported oral PSMP exposure levels in rodents associated with adverse biological effects [15,16,17]). PSMPs (particle diameter 0.5 μm) were prepared by diluting the stock suspension (10.13% solids content, according to the manufacturer’s specifications) 20-fold with ultrapure water and administered by oral gavage using a stainless-steel oral feeding needle (1.2 mm × 80 mm, ST-F174, Shin-Der, Taichung, Taiwan), with the gavage volume adjusted according to body weight (approximately 0.65–1.0 mL per administration). Administration was performed once every five days for a total of 15 administrations between day 0 and day 70.

Following the exposure period, animals were maintained for an additional 35-day recovery/observation period without further treatment to assess delayed cardiovascular and mitochondrial effects, as previous studies have demonstrated that microplastic-induced biological alterations may persist after exposure cessation [18]. Body weight was recorded weekly throughout the experiment.

On day 105, all animals were anesthetized with isoflurane and euthanized by exsanguination. Blood samples were collected from the abdominal aorta and centrifuged at 3000× *g* for 10 min at 4 °C for serum preparation. Organs (heart, liver, kidney, and spleen) were isolated, rinsed with physiological saline (0.9% NaCl) to remove residual blood, gently blotted dry, and weighed using an analytical balance (OHAUS PA214C, Parsippany-Troy Hills, NJ, USA) to determine both absolute and relative organ weights (normalized to the final body weight). In addition, the aorta was excised from the aortic root to the abdominal bifurcation under sterile conditions.

**Figure 1:**
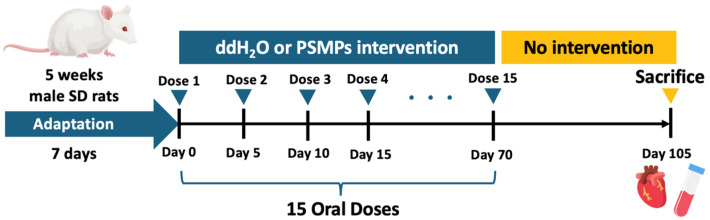
Experimental design and timeline of polystyrene microplastic exposure. Schematic illustration of the experimental design and exposure timeline. Male Sprague-Dawley rats were orally administered PSMPs every five days from day 0 to day 70 for a total of 15 doses. Following the final administration, animals were maintained without further exposure for an additional 35 days. All rats were sacrificed on day 105, and blood and cardiac tissues were collected for subsequent analyses.

The overall experimental design and sample allocation for downstream analyses are presented in Figure 1.

For downstream analyses, each heart was divided into two equal portions. One portion was used for biochemical and molecular analyses, including Western blotting, qPCR, and ELISA (*n* = 6 per group). The remaining portion was further divided for histological (H&E) and ultrastructural (TEM) analyses (*n* = 3 per group for each analysis).

To ensure accurate measurement, all organs were rinsed with 0.9% physiological saline to remove residual blood, gently blotted dry, and weighed using an electronic analytical balance (OHAUS Corporation, Parsippany-Troy Hills, NJ, USA; Model PA214C). Both absolute organ weight and relative organ weight (normalized to the final body weight) were recorded for analysis.

For subsequent tissue processing, each isolated heart (*n* = 6 per group) was randomly assigned to biochemical, histological, and ultrastructural analyses. Each heart was divided into two equal halves. One-half was used for biochemical and molecular analyses, including Western blotting, qPCR, and ELISA (*n* = 6 per group). The ventricular myocardium from the other half was equally divided for TEM and histological (H&E) analysis (*n* = 3 per group).

### 2.3. Biochemical Analysis

Serum samples were obtained by centrifugation (3000× *g* for 10 min at 4 °C) and analyzed using a fully automated clinical chemistry analyzer (Roche Modular P800, Roche Diagnostics GmbH, Mannheim, Germany). Aspartate aminotransferase (AST), alanine aminotransferase (ALT), blood urea nitrogen (BUN), creatinine (CRE), total cholesterol (TC), triglycerides (TG), low-density lipoprotein cholesterol (LDL-C), high-density lipoprotein cholesterol (HDL-C), and high-sensitivity C-reactive protein (Hs-CRP) were measured by colorimetric methods (Randox Laboratories Ltd., Crumlin, UK) according to the manufacturer’s instructions: AST (Cat. No. AS3804), ALT (Cat. No. AL3801), BUN (Cat. No. UR8334), CRE (Cat. No. CR4037), TC (Cat. No. CH3810), TG (Cat. No. TR3823), LDL-C (Cat. No. CH3841), HDL-C (Cat. No. CH203), and Hs-CRP (Cat. No. CP2476). Serum creatine kinase-MB (CK-MB) and cardiac troponin I (cTnI) levels were quantified using commercial ELISA kits (Elabscience Biotechnology Inc., Houston, TX, USA) according to the manufacturer’s instructions: CK-MB (Cat. No. E-EL-R1327) and cTnI (Cat. No. E-CL-R0721), with absorbance measured at 450 nm using an Epoch 2 Microplate Spectrophotometer (Agilent BioTek, Santa Clara, CA, USA). Serum lactate dehydrogenase (LDH) levels were determined using a commercial assay kit (ScienCell Research Laboratories, Carlsbad, CA, USA, Cat. No: 8078) according to the manufacturer’s instructions, with absorbance measured at 490 nm using the same instrument.

### 2.4. Oxidative Stress and Antioxidant Enzyme Activity

Oxidative stress markers and antioxidant enzyme activities were assessed in cardiac tissues and the aorta. Malondialdehyde (MDA) levels were measured using a thiobarbituric acid reactive substances (TBARS) assay kit (Cayman Chemical, Ann Arbor, MI, USA, Cat. No: 10009055), with absorbance measured at 530 nm. Tissues were homogenized in ice-cold buffer and subjected to differential centrifugation at 10,000× *g* for 15 min at 4 °C to obtain cytosolic and mitochondrial fractions. The supernatant was collected for cytosolic SOD1 activity analysis, whereas the mitochondrial pellet was resuspended in cold buffer (20 mM HEPES, pH 7.2, containing 1 mM EGTA, 210 mM mannitol, and 70 mM sucrose) for mitochondrial SOD2 activity measurement. All assays were performed according to the manufacturer’s instructions (Cayman Chemical, Ann Arbor, MI, USA, Cat. No: 706002), with absorbance measured at 530 nm. Catalase (CAT) activity was measured using a commercial assay kit (Cayman Chemical, Ann Arbor, MI, USA, Cat. No: 707002). All measurements were performed according to the manufacturer’s instructions and normalized to protein concentration, which was determined using a BCA assay kit (Visual Protein, Energenesis Co., Taipei, Taiwan, Cat. No: BC03-500). Absorbance was measured using an Epoch 2 Microplate Spectrophotometer (Agilent BioTek, Santa Clara, CA, USA).

### 2.5. Mitochondrial Complex Enzyme Activity

Mitochondrial respiratory chain enzyme activities were assessed in the cardiac tissue. Cardiac tissue (100 mg) was homogenized in Buffer 1 (1 mM EGTA, 250 mM sucrose, 10 mM HEPES, pH 7.4) at 4 °C. Mitochondria were isolated by differential centrifugation (600× *g* for 10 min followed by 7000× *g* for 15 min) and stored at −80 °C until analysis of nicotinamide adenine dinucleotide cytochrome c reductase (NCCR; complex I + III) activity and succinate cytochrome c reductase (SCCR; complex II + III) activity. Prior to assays, the mitochondrial pellet was resuspended in Buffer 2 (1 mM EDTA, 70 mM sucrose, 220 mM mannitol, 2 mM HEPES, pH 7.4). For NCCR measurement, mitochondrial extracts (20 μg protein) were incubated with 180 μL reaction buffer containing 1 mM NADH, 1.5 mM potassium cyanide, and 50 mM potassium phosphate buffer (pH 7.4). For SCCR measurement, mitochondrial extracts (20 μg protein) were incubated with 180 μL reaction buffer containing 25 mM succinate, 1.5 mM potassium cyanide, and 50 mM potassium phosphate buffer (pH 7.4). After incubation at 37 °C for 2 min, the reaction was initiated by adding 20 μL of 1 mM oxidized cytochrome c. Changes in absorbance at 550 nm were recorded over a 10 min period using an Epoch 2 Microplate Spectrophotometer (Agilent BioTek, Santa Clara, CA, USA). Enzyme activities were calculated and expressed as nmol of cytochrome c reduced per minute per milligram of protein.

### 2.6. Mitochondrial DNA Content Analysis

Total DNA was extracted from cardiac tissue using the DNeasy Blood and Tissue Kit (QIAGEN, Hilden, Germany, Cat. No: 69506) according to the manufacturer’s instructions. Mitochondrial DNA (mtDNA) content was measured by quantitative real-time PCR (qPCR) and normalized to nuclear DNA (nDNA) levels using β-actin (ACTB) as the nuclear reference gene. The qPCR conditions were as follows: initial denaturation at 95 °C for 10 min, followed by 40 cycles of denaturation at 95 °C for 15 s, annealing at 60 °C for 30 s, and extension at 72 °C for 30 s. Relative mtDNA copy number was calculated using the 2^−ΔCt^ method and expressed as fold change relative to the control group. qPCR was performed using mt-ND1 and ACTB-specific primers. The primer sequences (5′ → 3′) were: mt-ND1 forward TTAATTGCCATGGCCTTCCTCACC and reverse TGGTTAGAGGGCGTATGGGTTCTT; ACTB forward CCACCATGTACCCAGGCATT and reverse CGGACTCATCGTACTCCTGC [19].

### 2.7. Western Blotting

Frozen cardiac tissue samples were homogenized on ice in extraction buffer (0.2% SDS, 0.2% DOC, 1% Triton X-100, 50 mM Tris-HCl pH 7.6, 1 mM EDTA, 5% protease inhibitors), then 10,000× *g* for 15 min at 4 °C. Protein concentrations were determined using a BCA assay, and 30 µg of protein extracts were separated by SDS-PAGE and transferred to PVDF membranes (Cytiva, Marlborough, MA, USA, Cat. No: 10600023). Membranes were blocked with 5% bovine serum albumin (BSA) for 1 h at room temperature and incubated overnight at 4 °C with primary antibodies diluted in 5% BSA/TBST: PGC-1α (Proteintech, Cat#66369-1-Ig, 95 kDa, 1:1000), NRF-1 (Abcam, Cat#ab34682, 68 kDa, 1:1000), TFAM (Abcam, Cat#ab131607, 28 kDa, 1:2000), LC3 (Proteintech, Cat#14600-1-AP, 15–18 kDa, 1:2000), p62 (Proteintech, Cat#18420-1-AP, 62 kDa, 1:2000), Parkin (Abcam, Cat#ab15494, 54 kDa, 1:1000), PINK1 (Proteintech, Cat#23274-1-AP, 65 kDa, 1:2000), NOX4 (Proteintech, Cat#14347-1-AP, 67 kDa, 1:1000), and GAPDH (Proteintech, Cat#60004-1-Ig, 37 kDa, 1:20000). After washing, membranes were incubated with HRP-conjugated secondary antibodies: goat anti-mouse IgG (H + L) and goat anti-rabbit IgG (H + L) (Proteintech, Rosemont, IL, USA, Cat. No: RGAM001 and RGAR001; 1:4000), then developed with Enhanced Chemiluminescence (ECL) substrate SuperKine™ West Femto (Boster Biological Technology, Pleasanton, CA, USA, Cat. No: BMU102-EN) in the dark. Signals were captured using an Azure 280 system (Azure Biosystems, Dublin, CA, USA) and quantified with Visionworks software. Target protein expression was normalized to GAPDH.

### 2.8. Histological Analysis

Heart tissues were excised after sacrifice, rinsed with normal saline, and fixed in 10% neutral buffered formalin. Tissues were dehydrated, paraffin-embedded, sectioned, and stained with H&E according to standard histological procedures. Histological features were examined under a light microscope.

### 2.9. Transmission Electron Microscopy

Heart tissues from the ventricular myocardium were sectioned into approximately 1 mm^3^ pieces, fixed in 2.5% glutaraldehyde, post-fixed in 1% osmium tetroxide, dehydrated, embedded in epoxy resin, and sectioned for TEM examination (HT-7700, Hitachi, Tokyo, Japan). Mitochondrial morphology and myofibril structure were evaluated under TEM. Electron-dense particle-like structures observed in cardiac tissues were measured using ImageJ software (version 1.54g; National Institutes of Health, Bethesda, MD, USA).

### 2.10. Statistical Analysis

All data are presented as mean ± standard error of the mean (SEM). Statistical analyses were performed using GraphPad Prism (version 9.0; GraphPad Software, San Diego, CA, USA). Normality was assessed using the Shapiro–Wilk test, and homogeneity of variance was evaluated prior to statistical comparisons. One-way ANOVA followed by Tukey’s post hoc test was used for data meeting parametric assumptions, whereas Welch’s analysis of variance (ANOVA) followed by Games–Howell post hoc test was applied when assumptions were not satisfied. For within-group comparisons between baseline and day 105 body weights, paired *t*-tests were used. A *p*-value < 0.05 was considered statistically significant.

## 3. Results

### 3.1. Characteristics of PSMPs

**Figure 2:**
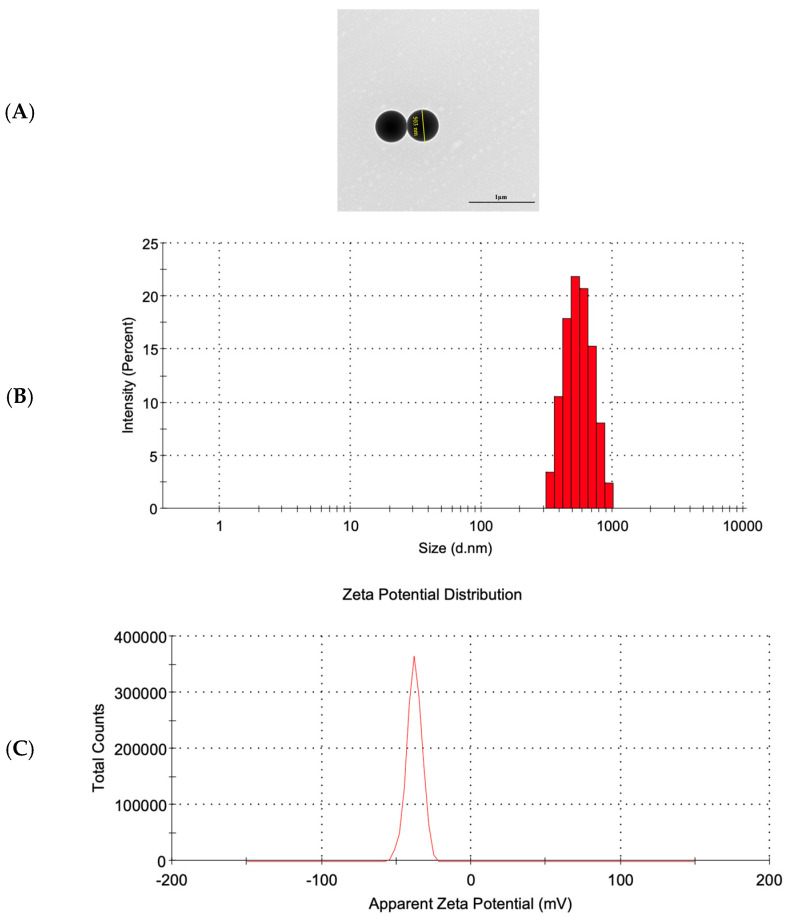
Characterization of polystyrene microplastics (PSMPs). (**A**) Representative transmission electron microscopy (TEM) image showing the morphology of PSMPs (scale bar = 1 µm; 7000× magnification) (**B**) Dynamic light scattering (DLS) analysis demonstrating the hydrodynamic diameter distribution of PSMPs in sterile distilled water. (**C**) Zeta potential analysis showing the surface charge of PSMPs.

Transmission electron microscopy (TEM) images demonstrated the morphology of PSMPs (Figure 2A). Dynamic light scattering (DLS) analysis in sterile distilled water showed a Z-average hydrodynamic diameter of 526.45 nm (Figure 2B). Zeta potential measurements indicated a surface charge of −37.8 mV (Figure 2C).

### 3.2. Effects of Microplastic Exposure on Body and Organ Weights

Body weights did not differ significantly among the control (C), low-dose PSMPs (L), and high-dose PSMPs (H) groups throughout the experimental period. Body weights increased progressively at 42, 75, and 105 days in all groups (Table 1A). At day 105, absolute and relative organ weights of the heart, liver, spleen, and kidney showed no significant differences among the three groups (Table 1B).

### 3.3. Effects of Microplastic Exposure on Serum Biochemical Parameters

To evaluate the systemic effects of PSMPs exposure, serum biochemical parameters related to hepatic function, renal function, lipid metabolism, inflammation, and cardiac alterations were assessed (Table 2).

Serum aspartate aminotransferase (AST), alanine aminotransferase (ALT), and the AST/ALT ratio showed no significant differences among groups. In contrast, serum blood urea nitrogen (BUN) levels were significantly increased in the low-dose PSMPs (L) group, whereas serum creatinine (CRE) levels were significantly elevated in the high-dose PSMPs (H) group.

Among lipid parameters, high-density lipoprotein cholesterol (HDL-C) levels were significantly reduced in the H group, while total cholesterol (TC), triglycerides (TG), and low-density lipoprotein cholesterol (LDL-C) levels did not differ significantly among groups. Serum high-sensitivity C-reactive protein (Hs-CRP) levels significantly increased in both PSMPs-exposed groups compared with the control group.

Selective changes in multiple cardiac biochemical markers were observed after exposure to PSMPs (Table 2). Serum lactate dehydrogenase (LDH) levels were significantly increased in the L group, while serum creatine kinase-MB (CK-MB) levels were significantly elevated in the H group. Although cardiac troponin I (cTnI) levels in the H group showed higher mean values compared with the control group, no statistically significant differences were detected among the groups.

### 3.4. Histopathological and Ultrastructural Alterations in Cardiac Tissue

**Figure 3:**
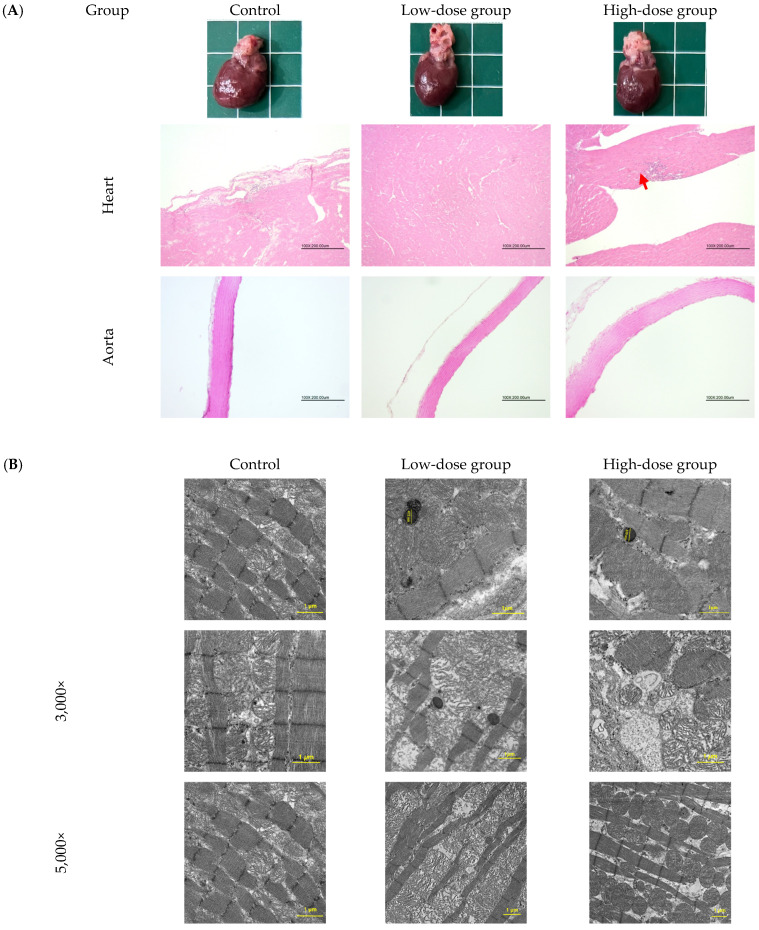
Histological and ultrastructural alterations in cardiac tissue following PSMPs exposure. (**A**) Representative gross morphology and H&E-stained sections of cardiac tissue from the control (C), low-dose PSMPs (L), and high-dose PSMPs (H) groups. No apparent differences in gross cardiac morphology were observed among groups. H&E staining showed preserved myocardial architecture in the C and L groups, whereas focal structural alterations were observed in the H group. (**B**) Representative TEM images of cardiomyocytes from each group. Cardiomyocytes in the C group exhibited normal ultrastructure. The L group showed mild ultrastructural changes, while the H group displayed more pronounced mitochondrial swelling, cristae disruption, and myofibrillar disorganization. Electron-dense particle-like structures were occasionally observed in PSMPs-exposed groups, and their size was evaluated in representative regions. Yellow lines indicate representative particle diameter measurements. Scale bars are indicated. C, control; L, low-dose PSMPs; H, high-dose PSMPs; H&E, hematoxylin and eosin; PSMPs, polystyrene microplastics; TEM, transmission electron microscopy (*n* = 3 per group).

Given the selective alterations observed in serum cardiac-related biomarkers, cardiac tissues were further examined to assess structural and ultrastructural changes associated with PSMPs exposure. H&E staining revealed preserved myocardial architecture in the control (C) group, characterized by regularly aligned cardiomyofibers and compact myocardial structure. In the low-dose PSMPs (L) group, myocardial morphology remained largely comparable to that of the control group, with no apparent widespread structural abnormalities. In contrast, cardiac sections from the high-dose PSMPs (H) group exhibited focal myocardial structural alterations (Figure 3A).

In the PSMPs-exposed groups, electron-dense particle-like structures were observed in cardiac tissues under TEM. The particle dimensions were measured in selected fields, with average sizes of approximately 420 nm and 472 nm in the L and H groups, respectively (Figure 3B). Cardiac tissue from the C group exhibited intact mitochondrial morphology with well-defined cristae and orderly myofibrillar organization. In comparison, cardiomyocytes from both the L and H groups showed mitochondrial swelling and disrupted cristae organization, accompanied by localized disorganization and loosening of myofibrillar alignment, indicating compromised ultrastructural integrity. Notably, these alterations were focal rather than uniform and were more evident in the H group (Figure 3B).

Histological examination of aortic sections revealed preserved vascular architecture across groups. No substantial intimal thickening, medial disruption, or inflammatory cell infiltration was observed following PSMPs exposure (Figure 3A).

### 3.5. Oxidative Stress and Antioxidant Defense in Cardiac and Aortic Tissues

**Figure 4:**
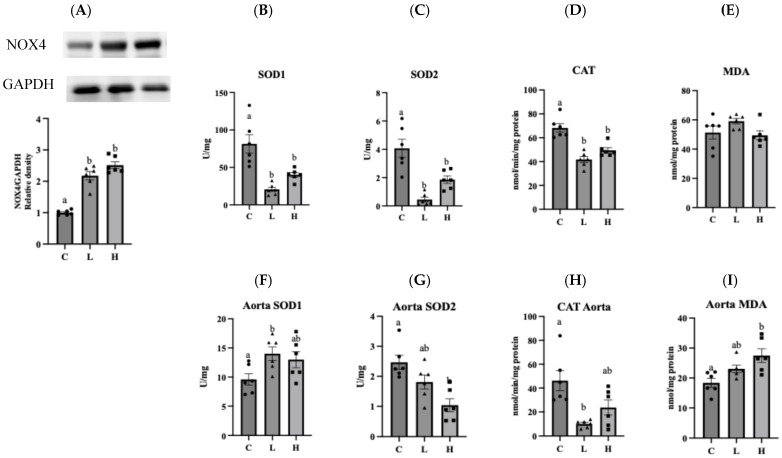
Effects of PSMPs exposure on oxidative stress-related markers and antioxidant enzyme activities in cardiac and aortic tissues. (**A**) Representative immunoblot images and quantitative analysis of cardiac NOX4 protein expression. Protein expression levels were normalized to GAPDH and expressed relative to the control group, (**B**) cardiac SOD1 activity, (**C**) cardiac SOD2 activity, (**D**) cardiac CAT activity, (**E**) cardiac MDA levels, (**F**) aortic SOD1 activity, (**G**) aortic SOD2 activity, (**H**) aortic CAT activity, (**I**) aortic MDA levels. Values are presented as the mean ± SEM (*n* = 6 per group). Mean values with different letters indicate statistically significant differences (*p* < 0.05), determined by one-way ANOVA followed by Tukey’s post hoc test. CAT, catalase; MDA, malondialdehyde; NOX4, NADPH oxidase 4; PSMPs, polystyrene microplastics; SOD1, superoxide dismutase 1; SOD2, superoxide dismutase 2; GAPDH, glyceraldehyde-3-phosphate dehydrogenase. C, control; L, low-dose PSMPs; H, high-dose PSMPs.

To further characterize oxidative stress status in cardiac tissue following PSMPs exposure, oxidative stress-related protein expression and antioxidant enzyme activities were evaluated (Figure 4).

Compared with the control group, cardiac NOX4 protein expression was significantly increased in both the L and H groups, with a greater elevation observed in the H group (Figure 4A).

In contrast, alterations in cardiac antioxidant defense were observed following PSMPs exposure. The activities of superoxide dismutase 1 (SOD1), superoxide dismutase 2 (SOD2), and CAT were significantly reduced in both the L and H groups compared with the C group (Figure 4B–D). Despite these changes, cardiac MDA levels did not differ significantly among the C, L, and H groups (Figure 4E). These findings prompted further investigation into mitochondrial respiratory function and mitochondrial-related alterations in cardiac tissue.

Compared with the control (C) group, aortic SOD1 and CAT activities were significantly increased in the low-dose (L) group (Figure 4F,H). In contrast, aortic SOD2 activity was significantly decreased, and MDA levels were significantly increased in the high-dose (H) group (Figure 4G,I).

### 3.6. Cardiac Mitochondrial Function

**Figure 5:**
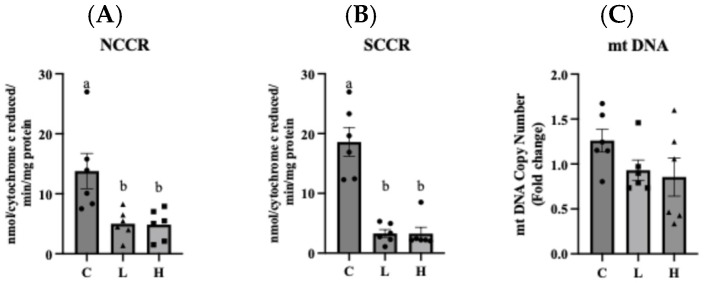
Effects of polystyrene microplastic exposure on cardiac mitochondrial respiratory chain activity and mitochondrial DNA content. (**A**) NCCR activity, (**B**) SCCR activity, (**C**) relative mitochondrial DNA. Values are presented as the mean ± SEM (*n* = 6 per group). Mean values with different letters indicate statistically significant differences (*p* < 0.05), determined by one-way ANOVA followed by Tukey’s post hoc test or Welch’s ANOVA with Games–Howell post hoc test, as appropriate. NCCR, NADH-cytochrome c reductase; PSMPs, polystyrene microplastics; SCCR, succinate-cytochrome c reductase. C, control; L, low-dose PSMPs; H, high-dose PSMPs.

To further evaluate whether PSMPs exposure was associated with alterations in cardiac mitochondrial function, the activities of mitochondrial respiratory chain complexes and mitochondrial DNA content were assessed in the cardiac tissue (Figure 5).

The activity of NCCR (complex I + III) was significantly reduced in both the low-dose PSMPs (L) and high-dose PSMPs (H) groups compared with the control (C) group (Figure 5A).

Similarly, SCCR (complex II + III) activity was significantly decreased in both the L and H groups relative to the C group (Figure 5B).

Notably, mitochondrial DNA (mtDNA) content in cardiac tissue did not differ significantly among the C, L, and H groups (Figure 5C).

### 3.7. Mitochondrial Quality Control in the Heart

**Figure 6:**
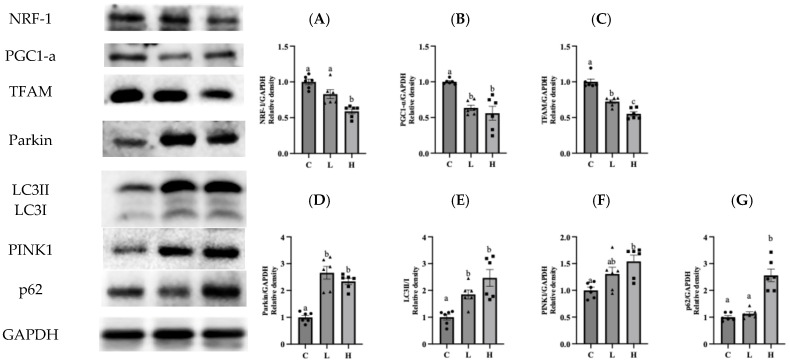
Alterations in mitochondrial biogenesis- and mitophagy-related proteins in cardiac tissue. Protein expression levels of mitochondrial biogenesis- and mitophagy markers in cardiac tissue from the control (C), low-dose PSMPs (L), and high-dose PSMPs (H) groups were analyzed by Western blotting: (**A**) NRF-1, (**B**) PGC-1α, (**C**) TFAM, (**D**) Parkin, (**E**) LC3-II/I ratio, (**F**) PINK1, and (**G**) p62. Representative immunoblot images are shown above the corresponding quantitative analyses. Protein expression levels were normalized to GAPDH and expressed relative to the control group. Values are presented as the mean ± SEM (*n* = 6 per group). Mean values with different letters indicate statistically significant differences (*p* < 0.05), determined by one-way ANOVA followed by Tukey’s post hoc test or Welch’s ANOVA with Games–Howell post hoc test, as appropriate. GAPDH, glyceraldehyde 3-phosphate dehydrogenase; LC3, microtubule-associated protein 1 light chain 3; NRF-1, nuclear respiratory factor 1; PGC-1α, peroxisome proliferator-activated receptor gamma coactivator 1-alpha; PINK1, PTEN-induced kinase 1; PSMPs, polystyrene microplastics; TFAM, mitochondrial transcription factor A. C, control; L, low-dose PSMPs; H, high-dose PSMPs.

To further examine whether alterations in mitochondrial respiratory function were accompanied by changes in mitochondrial quality control, proteins involved in mitochondrial biogenesis and mitophagy were analyzed in cardiac tissue (Figure 6).

Proteins associated with mitochondrial biogenesis exhibited differential alterations following PSMPs exposure. The protein expression level of peroxisome proliferator-activated receptor gamma coactivator 1-alpha (PGC-1α) was significantly reduced in both the low-dose PSMPs (L) and high-dose PSMPs (H) groups compared with the control (C) group. Nuclear respiratory factor 1 (NRF-1) expression was significantly decreased in the H group. In addition, mitochondrial transcription factor A (TFAM) expression was significantly reduced in both PSMPs-exposed groups relative to the control group (Figure 6A–C).

In contrast, proteins associated with mitophagy were upregulated following exposure to PSMPs. The protein levels of PTEN-induced kinase 1 (PINK1) and the adapter protein p62 were significantly increased in the H group compared with the C group, whereas the L group showed no significant changes. Parkin protein expression and the ratio of microtubule-associated protein 1 light chain 3-II to light chain 3-I (LC3-II/I) were significantly elevated in both the L and H groups relative to the C group (Figure 6D–G).

## 4. Discussion

The present study demonstrates that repeated exposure to PSMPs is associated with subclinical cardiovascular alterations characterized by redox imbalance, mitochondrial dysfunction, and altered mitochondrial quality control.

The dosing regimen used in the present study was designed based on both estimated human exposure and previously reported experimental models. Human microplastic intake has been estimated to range from approximately 0.1 to 5 g per week [1], supporting the use of a conservative low-dose exposure in experimental settings. Accordingly, the present study applied a dose gradient to enable assessment of potential dose-related cardiovascular and mitochondrial responses.

Previous studies have reported a broad range of plastic particle exposure doses (mainly polystyrene, PS), spanning from 0.01 mg/day to 250 mg/kg body weight/day, depending on the experimental purpose and exposure route [20]. This variability may be attributed to differences in study design and endpoints, as sub-chronic oral gavage studies generally use low- to moderate-dose (mg/kg) dosages to approximate environmentally relevant exposure levels or to investigate organ-specific responses [21,22,23].

Collectively, intermittent dosing regimens are commonly used in sub-chronic nanoplastic exposure studies to balance cumulative exposure and minimize stress associated with repeated gavage. Accordingly, dosing schedules in this field are generally designed in an intermittent manner rather than continuous daily exposure, depending on the specific experimental objectives and exposure models.

Under the present exposure conditions, repeated oral administration of 0.5 μm PSMPs at doses of 5 or 20 mg/kg did not result in significant changes in body weight, organ weight, or organ coefficients after exposure cessation. These measurements were included to evaluate whether exposure to PSMPs induced overt systemic toxicity beyond the cardiovascular system. Previous studies investigating the effects of micro- and nanoplastics on body weight have yielded inconsistent results, suggesting that systemic effects are highly dependent on exposure parameters, including particle size, dose, and duration. Larger microplastics (4–6 μm) have been associated with weight gain [24], whereas exposure to 0.5 μm particles has produced divergent outcomes depending on exposure duration, ranging from weight loss following prolonged exposure to no detectable changes after shorter exposure periods [25,26]. Collectively, these findings indicate that systemic weight-related alterations are not a consistent outcome of PSMPs exposure but are context-dependent and influenced by exposure design.

Despite the absence of significant alterations in serum total cholesterol and triglyceride levels during active exposure, a significant reduction in high-density lipoprotein cholesterol (HDL-C) was observed at the day 105 endpoint, particularly in the high-dose PSMPs group. This delayed lipid alteration suggests that repeated PSMPs exposure may induce subtle metabolic dysregulation that becomes detectable only after exposure has ceased. Similar delayed metabolic effects have been reported in animal models exposed to particulate air pollutants, including PM_2.5_ [27]. In agreement with the present findings, previous studies have demonstrated dose-dependent disruptions of lipid homeostasis following chronic PSMPs exposure [17]. Together, these observations suggest that PSMPs’ exposure may induce delayed changes in lipid metabolism rather than acute systemic dyslipidemia.

Although no overt systemic toxicity was detected at the day 105 endpoint, selective changes in serum cardiac-related biomarkers were observed, suggesting a possible subclinical cardiac response. Elevation of lactate dehydrogenase activity may reflect cellular stress rather than acute toxicity, whereas changes in CK-MB and cardiac troponin I were mild or transient, consistent with subclinical myocardial stress without evidence of extensive cardiomyocyte necrosis. Similar patterns of cardiac biomarker alterations have been reported following chronic PSMPs exposure, supporting the heart as a sensitive target organ under repeated exposure conditions [28,29]. A recent rat study using 0.5 μm PSMPs similarly reported cardiovascular biochemical alterations without obvious histopathological changes in the heart or aorta, consistent with the findings of the present study [17]. Notably, certain cardiac responses were more pronounced at lower exposure doses, consistent with non-monotonic dose–response relationships, including J-shaped patterns, which have been increasingly recognized in microplastic and endocrine-disrupting chemical research [30,31].

Because cardiomyocytes rely heavily on mitochondrial oxidative phosphorylation to sustain continuous contractile activity, they are particularly vulnerable to redox-mediated mitochondrial dysfunction under sustained oxidative stress conditions [32]. In the present study, NOX4 upregulation accompanied by reduced antioxidant enzyme activities, including SOD1, SOD2, and CAT, suggests a shift toward a pro-oxidative intracellular environment that may increase mitochondrial susceptibility to oxidative damage. This pattern may reflect a subthreshold oxidative disturbance rather than overt lipid peroxidation-mediated oxidative damage [33,34]. Previous studies have demonstrated that exposure to PSMPs can elicit oxidative and mitochondrial stress in a context-dependent manner. In hiPSC-derived kidney organoids, 1 μm PSMPs induced mitochondrial oxidative stress and B-cell lymphoma 2 (Bcl-2) family-mediated apoptosis [35]. In vivo, 5–10 μm PS-MPs have been shown to activate oxidative stress-related signaling cascades, including Toll-like receptor 4 (TLR4) and NADPH oxidase 2 (NOX2), leading to tissue structural alterations in mice [36]. However, inconsistent findings have also been reported, with no significant changes in classical oxidative stress markers observed in some rodent studies, potentially due to insufficient exposure intensity to elicit measurable oxidative damage [37]. Compared with these findings, the present results suggest that cardiac tissue may exhibit a redox-sensitive subclinical response to repeated 0.5 μm PSMP exposure, even in the absence of overt systemic toxicity.

The observed alterations suggest that exposure to PSMPs points to a possible redox–mitochondrial interaction, in which mitochondrial respiratory chain impairment may enhance reactive oxygen species production, while sustained redox imbalance further compromises mitochondrial function. In this context, NOX4 is a possible redox-associated modulator, not the central pathway [38]. This interaction may underlie sustained mitochondrial dysfunction associated with delayed cardiovascular alterations.

Concurrent impairment of mitochondrial respiratory chain function was evidenced by significantly reduced NCCR (complex I + III) and SCCR (complex II + III) activities. Complex I/III dysfunction may amplify ROS production and maintain a redox–mitochondrial feedback loop [39,40]. Excessive ROS may further disrupt mitochondrial membrane potential, thereby establishing a feed-forward cycle of mitochondrial dysfunction. This redox–mitochondrial interaction may contribute to the mitochondrial impairment observed following exposure [41].

NOX4 has been reported to localize to mitochondria and interact with complex I, suggesting a potential link between NOX4-mediated redox regulation and mitochondrial respiratory function. In this study, NOX4 upregulation was observed in parallel with impaired mitochondrial activity, indicating a possible association within the redox–mitochondrial network. Consistent with this concept, previous studies have suggested that NOX4 activation may participate in a redox–mitochondrial feedback network involving mitochondrial dysfunction, endoplasmic reticulum (ER) stress, and dysregulated mitophagy [42]. Environmental particulate exposure may induce NOX4-dependent redox imbalance and mitochondrial damage, further supporting the presence of a redox–mitochondrial feedback network [43]. Together, these findings suggest that NOX4 may be involved in the PSMPs-related redox–mitochondrial response, although its precise mechanistic contribution requires further validation.

To determine whether mitochondrial dysfunction was accompanied by alterations in mitochondrial maintenance pathways, we further examined mitochondrial quality-control mechanisms. In PSMPs-exposed hearts, key regulators of mitochondrial biogenesis, including PGC-1α, NRF-1, and TFAM, remained suppressed after exposure cessation, suggesting impaired mitochondrial biogenesis-related signaling. In parallel, alterations in mitophagy markers were observed, including changes in PINK1/Parkin expression, increased LC3-II/I ratio, and accumulation of p62. Previous studies have shown that oxidative stress negatively regulates the PGC-1α/NRF-1 signaling axis in cardiovascular tissues, thereby limiting compensatory mitochondrial biogenesis under chronic stress conditions [32]. Moreover, the concurrent elevation of LC3-II and p62 suggests impaired autophagic flux as well as inefficient clearance of dysfunctional mitochondria [44,45].

Therefore, mitochondrial dysfunction in PSMPs-exposed hearts may be associated with redox imbalance, respiratory chain impairment, and defective mitochondrial quality control.

Aortic tissue was analyzed to assess whether PSMPs’ exposure may also influence vascular redox-related parameters in addition to cardiac alterations. Although aortic tissue did not show overt histopathological remodeling, several oxidative stress-related parameters were altered, suggesting that the vascular response may have been in a subclinical or pre-remodeling phase at day 105. Previous studies have indicated that the structural robustness and relatively low metabolic demand of large elastic arteries may mean that short-term or moderate oxidative stress is insufficient to induce detectable medial or intimal remodeling [46]. Therefore, compared with cardiomyocytes, vascular tissue may exhibit relatively lower susceptibility to early structural alterations under the present exposure conditions.

Several limitations should be acknowledged. First, the lack of data at time point day 70 limits the assessment of the effects of time recovery. Second, the proposed NOX4-related mechanisms could not be fully validated in this experiment. Third, this study did not assess mitochondrial dynamics because fusion and fission-related proteins (such as Drp1, Mfn1/2, and OPA1) were not included. Fourth, only male rats were used in this study; therefore, potential sex-related differences in the cardiovascular response to PSMPs exposure could not be evaluated. Future research should incorporate NOX4 inhibitors or antioxidant interventions to elucidate their mechanistic effects and assess mitochondrial dynamics.

Despite these limitations, the present study provides integrated evidence that PSMPs’ exposure induces subclinical cardiovasular alterations associated with redox imbalance and mitochondrial dysfunction, with delayed cardiovascular effects.

## 5. Conclusions

Exposure to PSMPs resulted in sustained redox imbalance, impaired mitochondrial respiratory chain activity, ultrastructural mitochondrial abnormalities, and dysregulation of mitochondrial quality control, hence supporting a state of chronic, moderate oxidative stress rather than acute lipid peroxidation-mediated injury. In contrast, although oxidative stress-related markers were elevated in the aorta, no overt structural remodeling was observed, suggesting that vascular alterations may remain at a subclinical or pre-remodeling stage. Collectively, these findings suggest that exposure to PSMPs is associated with cardiac mitochondrial alterations at the molecular level and suggests potential risks to cardiovascular health.

## Figures and Tables

**Table 1:** Body weight and organ weight assessments.

(**A**)						
**Group**	**C**		**L**		**H**	
Initial weight (g)	144.9 ± 4.3		143.7 ± 2.5		143.6 ± 2.7	
42 days weight (g)	375.5 ± 5.3		385.3 ± 8.7		389.7 ± 8.7	
75 days weight (g)	488.1 ± 6.5		479.5 ± 4.8		487.2 ± 3.4	
105 days weight (g)	531.6 ± 4.3 *		526.1 ± 7.6 *		545.9 ± 12.2 *	
(**B**)						
**Group**	**C**		**L**		**H**	
	OW (g)	% BW	OW (g)	% BW	OW (g)	% BW
Heart	1.59 ± 0.09	0.31 ± 0.02	1.66 ± 0.08	0.31 ± 0.02	1.65 ± 0.14	0.3 ± 0.03
Liver	11.51 ± 1.14	2.23 ± 0.1	12.42 ± 1.02	2.32 ± 0.12	13.13 ± 1.91	2.4 ± 0.1
Spleen	0.64 ± 0.1	0.13 ± 0.02	0.65 ± 0.09	0.12 ± 0.01	0.7 ± 0.11	0.13 ± 0.02
Kidney	3.19 ± 0.15	0.62 ± 0.03	3.43 ± 0.28	0.64 ± 0.04	3.35 ± 0.28	0.62 ± 0.05

(A) Values are presented as the mean ± SEM (*n* = 6 per group). No significant differences were observed among groups at each time point, as determined by one-way ANOVA followed by Tukey’s post hoc test. * *p* < 0.05 versus initial weight within the same group at day 105, determined by paired *t*-test. C, control; L, low-dose PSMPs; H, high-dose PSMPs; (B) Values are presented as the mean ± SEM (*n* = 6 per group). No statistically significant differences were observed among groups, as determined by one-way ANOVA followed by Tukey’s post hoc test. C, control; L, low-dose PSMPs; H, high-dose PSMPs; OW, organ weight; % BW, percentage of body weight.

**Table 2:** Effects of polystyrene microplastic exposure on serum biochemical parameters.

	C	L	H
AST (U/L)	79.2 ± 4.16	95.8 ± 8.12	90.2 ± 3.18
ALT (U/L)	32.5 ± 1.59	33.2 ± 2.78	39.3 ± 1.55
AST/ALT ratio	2.4 ± 0.12	3.0 ± 0.29	2.3 ± 0.04
BUN (mg/dL)	13 ± 0.16 ^a^	14.5 ± 0.37 ^b^	14.1 ± 0.37 ^ab^
CRE (mg/dL)	0.2 ± 0.01 ^a^	0.2 ± 0.02 ^ab^	0.3 ± 0.02 ^b^
TC (mg/dL)	44.0 ± 0.98	47.5 ± 3.06	51.6 ± 4.20
TG (mg/dL)	41.3 ± 2.04	46.6 ± 3.55	50.3 ± 3.88
HDL-C (mg/dL)	21.2 ± 0.78 ^a^	18.3 ± 0.90 ^ab^	17.8 ± 0.94 ^b^
LDL-C (mg/dL)	4.0 ± 0.00	4.0 ± 0.24	4.3 ± 0.20
Hs-CRP (ng/mL)	21.7 ± 3.06 ^a^	38.3 ± 3.06 ^b^	36.7 ± 2.12 ^b^
cTnI (pg/mL)	120.6 ± 13.55	125.6 ± 26.25	163.0 ± 13.68
CK-MB (pg/mL)	534.8 ± 69.83 ^a^	641.9 ± 151.10 ^ab^	989.2 ± 72.78 ^b^
LDH (%)	2513.3 ± 214.76 ^a^	5908.8 ± 655.88 ^b^	4090.7 ± 796.81 ^ab^

Values are presented as the mean ± SEM (*n* = 6 per group). Mean values with different letters indicate statistically significant differences (*p* < 0.05), determined by one-way ANOVA with Tukey’s post hoc test or Welch’s ANOVA with Games–Howell post hoc test, as appropriate. ALT, alanine aminotransferase; AST, aspartate aminotransferase; BUN, blood urea nitrogen; CK-MB, creatine kinase-MB; CRE, creatinine; cTnI, cardiac troponin I; HDL-C, high-density lipoprotein cholesterol; Hs-CRP, high-sensitivity C-reactive protein; LDH, lactate dehydrogenase; LDL-C, low-density lipoprotein cholesterol; TC, total cholesterol; TG, triglycerides. C, control; L, low-dose PSMPs; H, high-dose PSMPs.

## Data Availability

Data are contained within the article/Supplementary Materials.

## Supplementary Materials

The following supporting information can be downloaded at: https://www.mdpi.com/article/10.3390/antiox15070816/s1, Figure S1: The original western blot images.

## Abbreviations

The following abbreviations are used in this manuscript:

ALT	Alanine aminotransferase
ANOVA	Analysis of variance
AST	Aspartate aminotransferase
ATP	Adenosine triphosphate
BCA	Bicinchoninic acid
BSA	Bovine serum albumin
Bcl-2	B-cell lymphoma 2
BUN	Blood urea nitrogen
CAT	Catalase
CK-MB	Creatine kinase-MB
CRE	Creatinine
cTnI	Cardiac troponin I
DLS	Dynamic light scattering
ECL	Enhanced chemiluminescence
ER	endoplasmic reticulum
GAPDH	Glyceraldehyde 3-phosphate dehydrogenase
HDL-C	High-density lipoprotein cholesterol
H&E	Hematoxylin and eosin
Hs-CRP	High-sensitivity C-reactive protein
IACUC	Institutional Animal Care and Use Committee
LC3	Microtubule-associated protein 1 light chain 3
LDH	Lactate dehydrogenase
LDL-C	Low-density lipoprotein cholesterol
MDA	Malondialdehyde
MQC	Mitochondrial quality control
mtDNA	Mitochondrial DNA
NCCR	Nicotinamide adenine dinucleotide cytochrome c reductase (complex I + III)
NOX4	NADPH oxidase 4
NRF-1	Nuclear respiratory factor 1
Nrf2	Nuclear factor erythroid 2-related factor 2
p62	Sequestosome 1
Parkin	Parkin RBR E3 ubiquitin protein ligase
PGC-1α	Peroxisome proliferator-activated receptor gamma coactivator 1-alpha
PINK1	PTEN-induced kinase 1
PSMPs	Polystyrene microplastics
PVDF	Polyvinylidene difluoride
qPCR	Quantitative real-time polymerase chain reaction
ROS	Reactive oxygen species
SCCR	Succinate cytochrome c reductase (complex II + III)
SDS-PAGE	Sodium dodecyl sulfate-polyacrylamide gel electrophoresis
SOD1	Superoxide dismutase 1
SOD2	Superoxide dismutase 2
TBARS	Thiobarbituric acid reactive substances
TEM	Transmission electron microscopy
TFAM	Mitochondrial transcription factor A
TG	Triglycerides
TC	Total cholesterol
HRP	Horseradish peroxidase
SEM	Standard error of the mean
