# Supplementary material for: Polystyrene Microplastics Induce Sustained Cardiovascular Redox Imbalance and Alter Mitochondrial Quality Control
Source: Antioxidants (Basel). 2026 Jun 29;15(7):816. doi: 10.3390/antiox15070816 (PMC13405854; doi:10.3390/antiox15070816)
Supplement: Supplementary file 1 [file antioxidants-15-00816-s001.zip › antioxidants-4292411-supplementary.pdf]

Figure S1. The original western blot images

NOX4_67 kDa

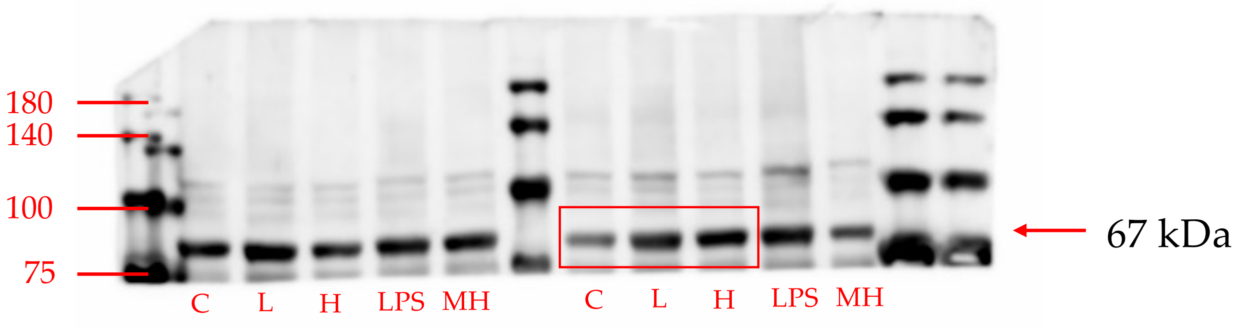


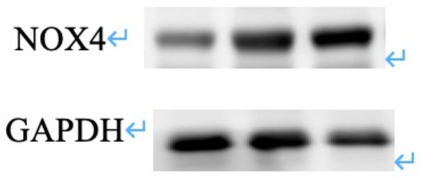


GAPDH_37 kDa

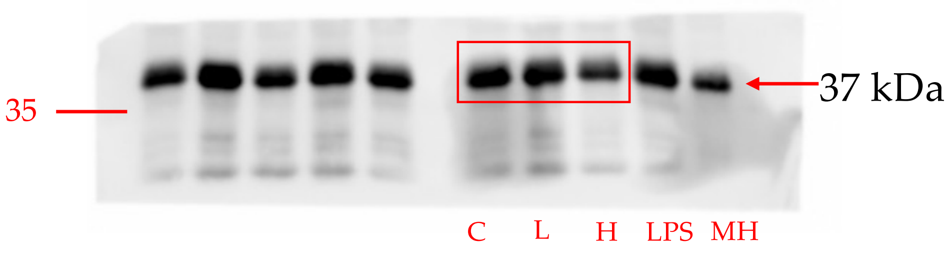

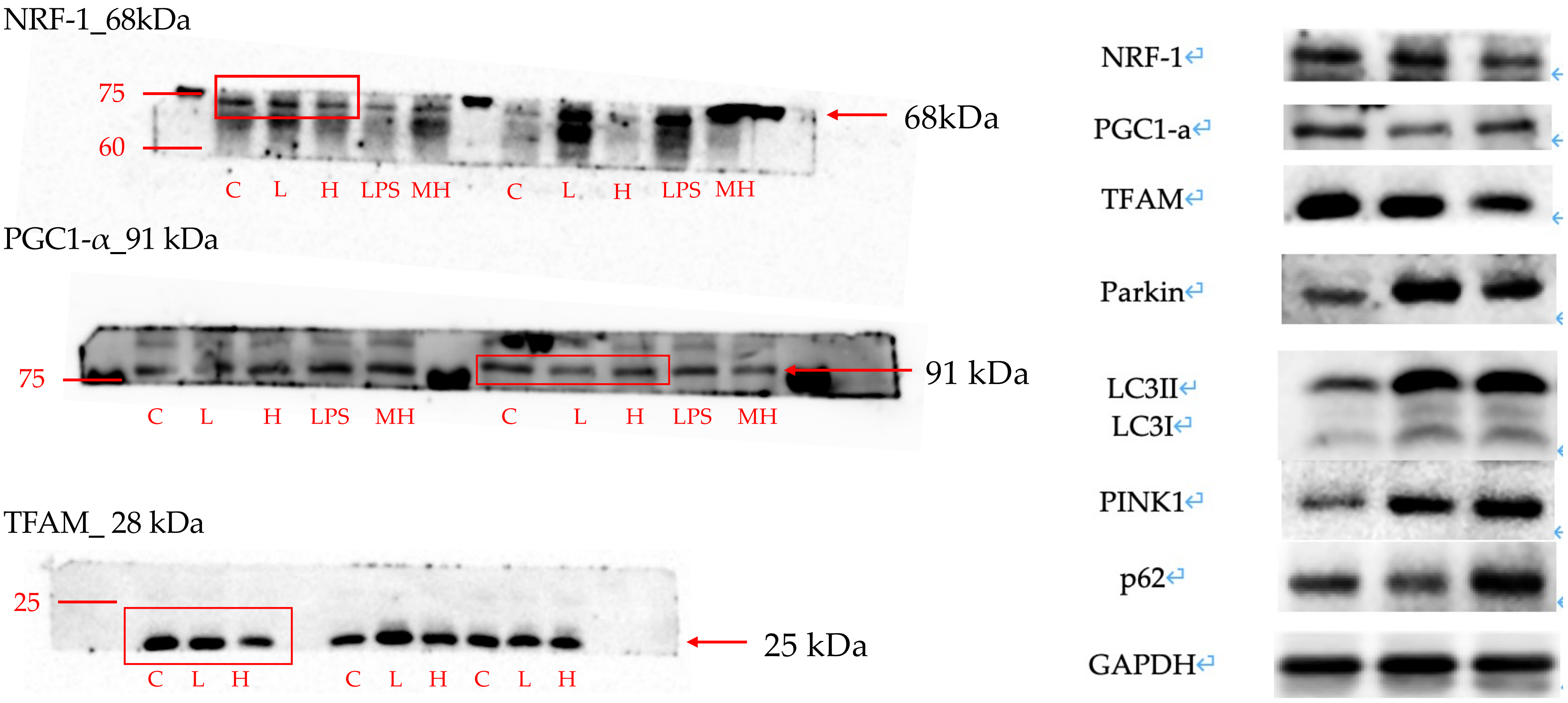

NRF-1_68kDa
75
60
C L H LPS MH C L H LPS MH
68kDa
PGC1-α_91 kDa
75
C L H LPS MH C L H LPS MH
91 kDa
TFAM_ 28 kDa
25
C L H C L H C L H
25 kDa
NRF-1
PGC1-a
TFAM
Parkin
LC3II
LC3I
PINK1
p62
GAPDH

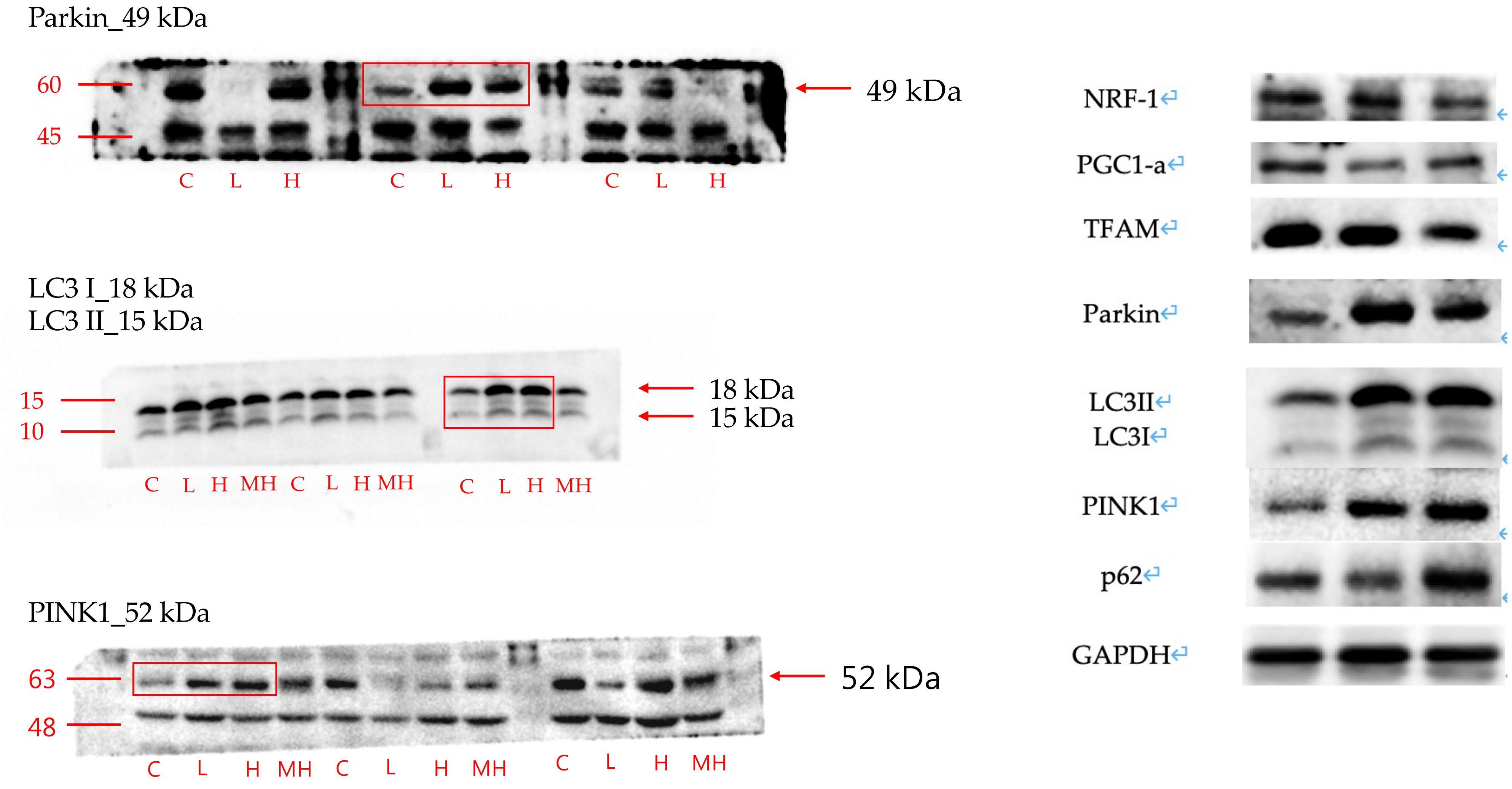
Parkin_49 kDa
60
45
49 kDa
C L H C L H C L H
LC3 I_18 kDa
LC3 II_15 kDa
15
10
18 kDa
15 kDa
C L H MH C L H MH C L H MH
PINK1_52 kDa
63
48
52 kDa
C L H MH C L H MH C L H MH
NRF-1
PGC1-a
TFAM
Parkin
LC3II
LC3I
PINK1
p62
GAPDH

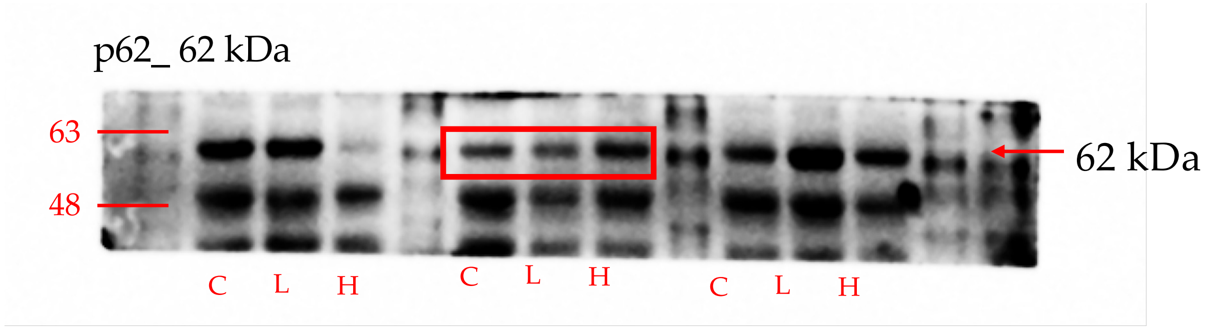
p62_ 62 kDa
63
48
62 kDa
C L H C L H C L H

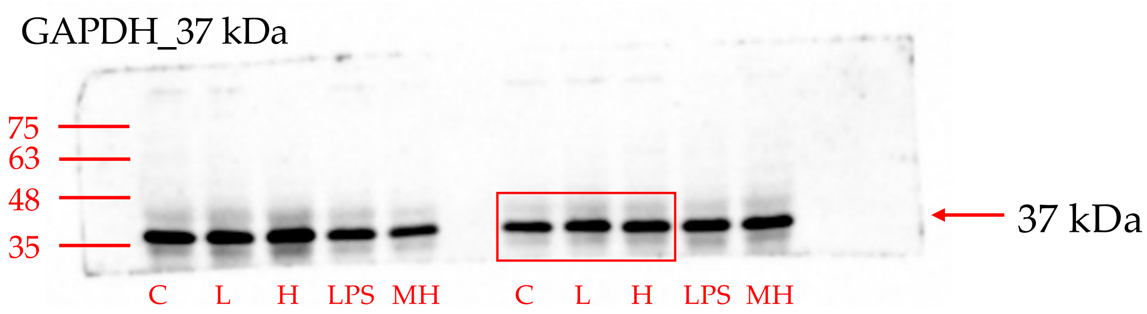
GAPDH_37 kDa
75
63
48
35
37 kDa
C L H LPS MH C L H LPS MH

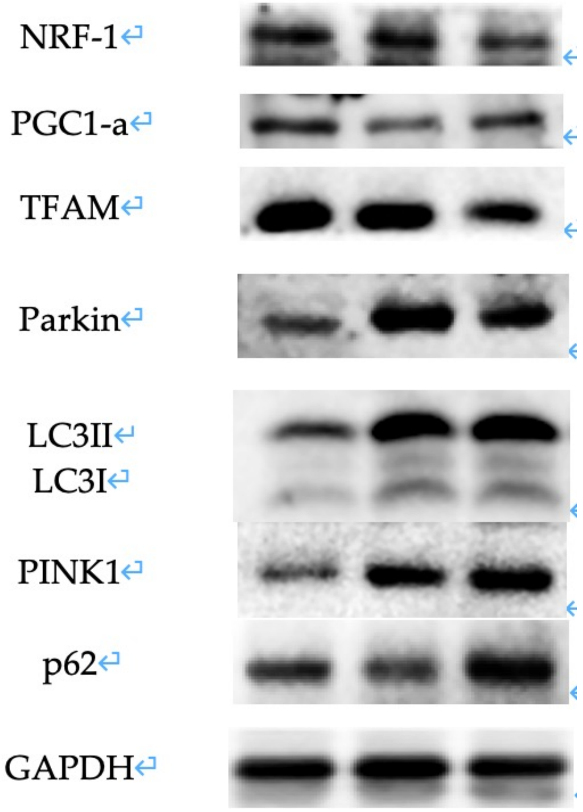
NRF-1
PGC1-a
TFAM
Parkin
LC3II
LC3I
PINK1
p62
GAPDH
